# Art therapy in addiction recovery: the face of addiction through artworks based on clinical case studies

**DOI:** 10.1186/s13722-026-00706-x

**Published:** 2026-07-30

**Authors:** Petra Arnold, Máté Kapitány-Fövény

**Affiliations:** 1 Department of Addictology, Faculty of Health Sciences, Semmelweis University, Vas u. 17, Budapest, 1088 Hungary; 2 Nyírő Gyula National Institute of Psychiatry and Addictions, Budapest, Hungary

**Keywords:** Addiction, Art therapy, Recovery, Clinical case study

## Abstract

Given the multifaceted nature of addiction, a multidisciplinary and comprehensive therapeutic approach is essential. Art therapy has therefore become an important complementary intervention in both inpatient and outpatient treatment settings. Numerous studies support its applicability and effectiveness among individuals with addiction. Art therapy can be particularly beneficial in reducing pervasive feelings of shame and anxiety, as well as in facilitating the processing of traumatic experiences - phenomena that are common among people with substance use disorder (SUD) and are often difficult to articulate verbally. The study examines the use of art therapy with clients with SUD, with a specific focus on the therapeutic factors that emerge through clinical practice. The analysis is grounded in three interrelated theoretical frameworks that help explain potential mechanisms of change in art therapy: trauma theory, mentalization-based approaches, and neurobiological and reward-related models. The case studies draw on two closed art therapy groups, consisting of 8 and 12 sessions respectively, at a drug outpatient treatment centre. Each group comprised eight adult participants with SUD. Most participants presented with comorbid conditions. Findings from the case studies suggest that the therapeutic factors associated with art therapy play a meaningful role in clients’ recovery processes. Evidence of recovery is reflected in the artworks produced during therapy, which offer insight into clients’ personalities, emerging behavioural and emotional patterns, and ongoing personal transformation. These visual expressions reflect clients’ current level of self-awareness and may highlight obstacles that may hinder recovery. In this way, the artworks function as visual narratives that both document and support the therapeutic process. The findings provide practical implications for clinicians seeking to integrate art therapy into addiction treatment. They also contribute to a deeper understanding of the therapeutic potential of the arts in supporting recovery among individuals with SUD. Given the high prevalence of comorbid personality disorders and trauma histories among individuals with SUD, art therapy serves as a uniquely adaptable modality that complements both verbal psychotherapy and pharmacological interventions. Art therapy is also a valuable tool for status assessment, offering insight into personality traits and emotional states.

## Clinical trial number

Not applicable.

## Introduction

Given the complex nature of addiction, a multidisciplinary therapeutic approach is essential for effective treatment. Consequently, nonverbal methods such as art therapy are frequently integrated as complementary interventions in both inpatient and outpatient clinical settings [1]. Several studies have confirmed the applicability and effectiveness of this approach among individuals with substance use disorder (SUD) [1, 2]. The act of creation can assist clients in articulating their emotions, processing them, and thereby initiating personal change and development [3].

Art therapy serves as a powerful tool for individuals with SUD, enabling them to manage overwhelming emotions such as shame and anxiety. It also facilitates the processing of trauma - often deeply rooted and difficult to verbalise - by providing a nonverbal outlet for expression and healing [1, 4]. These profound emotions and experiences often emerge more readily through creative expression than through verbal communication. The creative process allows clients to externalise and process their inner experiences, thereby fostering symbolic transformation within the therapeutic context [3].

Recent research further supports the potential efficacy of art therapy in trauma processing, highlighting its ability to activate neural pathways affected by trauma and its effectiveness in engaging individuals with cognitive impairments through experiential methods [5]. Art therapy contributes to emotional and impulse regulation, strengthens and stabilises identity, enhances emotional awareness and expression, and supports the acceptance of personal limitations and vulnerabilities.

Beyond its widespread clinical use, the effectiveness of art therapy has been increasingly examined in empirical research. A comprehensive systematic review by Regev and Cohen‑Yatziv [6], synthesizing quantitative outcome studies published between 2000 and 2017, identified a growing number of higher‑quality investigations, including randomized controlled trials, across diverse adult populations. These studies provide evidence that art therapy may contribute to improvements in emotional well‑being, quality of life, coping resources, and selected symptom domains in specific clinical contexts, such as medical illness, trauma exposure, and aging. At the same time, the review highlights substantial heterogeneity in study designs, outcome measures, and target populations, as well as mixed findings in certain areas (e.g., severe mental illness), underscoring that art therapy effects are neither universal nor uniform.

## Background of art therapy

The development of art therapy was influenced by several key factors, including the theories of Freud and Jung, psychiatric collections showcasing patients’ artwork, the emergence of art brut and outsider art, as well as movements such as expressionism and surrealism [7, 8]. Initially, the artworks of psychiatric patients were approached from distinct medical and aesthetic perspectives [7]. From the mid-20th century onward, the establishment of art therapy schools and professional societies facilitated its integration into various domains of healthcare and mental hygiene.

Art therapy has since emerged as a specialised discipline with its own body of knowledge and methodologies. It is a nonverbal therapeutic approach widely applied in clinical psychiatry, education, social work, and special education. According to the American Art Therapy Association, “Art therapy is a mental health profession that enriches the lives of individuals, families, and communities through active artmaking, creative process, applied psychological theory, and human experience within a psychotherapeutic relationship” [9]. The primary aim of art therapy is therapeutic rather than aesthetic or diagnostic; it employs structured methods and techniques to foster personal growth and psychological transformation [7, 10].

Margaret Naumburg [11] was the first to integrate art therapy with Freudian psychoanalysis, thereby establishing the foundation for art psychotherapy. As a result, a distinction is now made between art psychotherapy and art therapy, although they are often used in tandem with differing emphases. Art psychotherapy incorporates artistic and creative activities into the psychotherapeutic process, focusing on the interpretation and discussion of the artwork’s content [11]. In contrast, art therapy emphasises the process of creation itself, which is considered the central healing mechanism [12]. The therapist serves as a facilitator, supporting the creative process and offering emotional guidance. Rather than interpreting symbolic content, the focus is on the emotions and experiences elicited by the act of creation.

Kramer [12] asserts that “Art therapy is seen as distinct from psychotherapy. Its healing potentialities depend on psychological processes that are activated in creative work” (p. 25). The artwork functions as a container for unconscious conflicts, allowing the creator to engage with instinctual and emotional content that may be difficult to articulate verbally. Through spontaneous expression, individuals externalise inner experiences onto paper. In the subsequent verbal phase, group interactions and associations help participants gain insight into their internal processes [10, 12]. The therapeutic group itself may also serve as a symbolic “container” or “mother,” with the therapist’s communication reflecting the nurturing role of a caregiver. In this way, art therapy contributes directly to the reconstruction and reinforcement of the individual’s sense of self [13].

## Art therapy among individuals with SUD/AUD

Art therapy was first employed with individuals with SUD in the United States during the 1950s [14]. In Hungary, art therapy methods emerged later, in the 1960s and 1970s. This therapeutic approach is particularly effective in alleviating dominant emotions commonly experienced by individuals with SUD - such as shame and anxiety - and in facilitating the processing of traumatic experiences. Reducing shame is essential to the recovery process, as it can obscure and disrupt treatment if not properly identified and addressed [15]. Shame may manifest in the form of silence, anger, confusion, and projection [16]. Individuals in early stages of treatment often struggle to recognise feelings of shame [15]. High‑quality empirical studies, including randomized controlled trials, have provided evidence supporting the effectiveness of art therapy in addressing deep, and unspoken emotional pain and shame, as well as reducing symptoms of depression and impulsivity among patients with alcohol use disorder (AUD) [1, 17, 18]. A series of studies have confirmed its anxiety-reducing effects [19–22].

In addition to shame and anxiety, traumatisation is prevalent among individuals with SUD [23, 24]. Compulsive behaviours often arise as maladaptive strategies for self-regulating traumatic memories [3, 25]. Although traumatic experiences may be suppressed and memories forgotten, the associated pain continues to accumulate [15]. Traumatic memories often surface more readily through images than through verbal expression, as visual creation can bypass cognitive defences and access deeper emotional layers. Art offers both security and emotional distance from traumatic content through metaphorical and symbolic representation, while simultaneously allowing for full expression of the experience. Preverbal traumas may emerge in the artwork, and art therapy facilitates deeper exploration of emotional memory and early experiences. The unspeakable can be expressed, while safety and support are maintained throughout the creative process [15].

Art therapy contributes to reducing denial [26] by providing a non-threatening, symbolic mode of expression that bypasses rigid verbal defences - through creative processes, clients externalize internal conflicts, making them more accessible for reflection and integration -, lowering resistance to treatment [27], expressing emotions, dismantling defence mechanisms, enhancing executive functions, managing anger, improving self-regulation, increasing self-esteem, and alleviating feelings of isolation [28]. Its effectiveness is also observable at the neurobiochemical level [5]: research has shown that, like the effects of certain drugs, the activation of dopamine pathways during creative activity induces pleasurable sensations [29]. For individuals in recovery, this presents a significant therapeutic advantage - art therapy enables access to rewarding emotional states in a sober, substance-free manner [4, 30]. It is crucial to help clients recognise that creativity is an inherent aspect of their personality - one that can be accessed and nurtured independently of substance use [4, 10].

Art helps individuals with SUD access and express emotions and identify internal resources. It transforms energies dulled by substance use into creative expression, supports the recovery process, and reflects the client’s progress. Art therapy also helps maintain boundaries (e.g., group duration, paper size, available tools, and time allocated for artwork) while preserving the freedom to create and express emotions. Clients must make decisions about tools, colours, and techniques, which fosters a sense of control, self-regulation, competence, independence, and agency - without the need for self-medication - through the completion of their artwork [4].

Group art therapy creates a supportive environment where individuals with SUD can freely express thoughts and feelings - something they may not have been able to do in dysfunctional family systems [4, 15]. Through creativity, they experience attention and understanding. Group therapy offers a communal experience, reduces isolation, shame, and stigmatisation, and provides support, resources, creativity, acceptance, and opportunities for connection [1, 4, 31]. The group’s sustaining power mitigates loneliness and helps clients experience connection within a new relational system. Simultaneously, the solitary nature of creative work fosters self-affirmation: clients realise they are capable of creating something independently and experiencing their uniqueness, which strengthens self-connection [32]. The artwork functions as a transitional object onto which clients project negative emotions, offering a surface for expression. Difficult emotions and anxiety arising during the creative process can be alleviated by sharing with the group, especially through peer associations and recognition of shared experiences [4, 10]. Feedback from group members is often more readily accepted when the artwork confronts the creator with problematic patterns as tangible “evidence” [1]. Association and reflection on the artwork promote interpersonal connection, facilitate group communication [33], and support quieter clients who may struggle with symbolic content, helping them interpret images and engage more deeply [2]. This process can motivate behavioural change [34], moving clients from reflection to action [35].

Art therapy offers numerous benefits beyond addiction treatment [10, 36, 37]. Through its imaginative and nonverbal techniques, it may provide forms of expression that are less consciously controlled and, for some individuals, experienced as less susceptible to deliberate manipulation or resistance. Art‑based processes can support the externalization and symbolic representation of emotional experiences, allowing these materials to be revisited and reflected upon within the therapeutic relationship. In this way, art‑making has been conceptualized as a bridge between internal emotional states and external expression, facilitating engagement with preverbal or difficult‑to‑verbalize content.

## Goal and method

Art therapy has demonstrated a wide range of powerful therapeutic effects; however, the existing literature contains relatively few studies examining its application among individuals with SUD. The present study seeks to contribute to filling this gap. This study presents case studies to illustrate how key elements (therapeutic effects of art therapy) of art therapy manifest in therapeutic work with clients with SUD. It explores how typical symptoms, behavioural tendencies, and cognitive patterns associated with addiction - as well as the stages of recovery - are reflected in clients’ artistic expressions.

To deepen the interpretative framework of the presented case studies, the analysis will be situated within three interrelated theoretical perspectives that elucidate the potential mechanisms of change in art therapy. First, trauma theory [3, 25] provides a lens for understanding how creative processes offer a safe, symbolic container for the externalization and gradual integration of traumatic experiences, often inaccessible through verbal means. Second, the mentalization-based approach [38] highlights the role of art therapy in fostering reflective functioning and affect regulation, enabling clients to transform implicit emotional states into representational forms that can be verbalized and integrated within the therapeutic dialogue. Third, neurobiological and reward-related models [5, 30] underscore the activation of dopaminergic pathways during creative engagement, positioning art-making as a ‘sober reward system’ that supports resilience and adaptive coping. These theoretical pillars will guide the interpretation of therapeutic factors observed in the case material, including shame reduction, trauma processing, emotional regulation, and identity reconstruction within a structured group context.

The concept of analysis is as follows: the evaluative process integrates both manifest and latent dimensions of the artwork. Manifest analysis focuses on observable, concrete elements such as the client’s choice of materials, the structural composition, and the formal qualities of the image. Latent analysis, in contrast, explores the underlying psychological processes reflected in the artwork, including the client’s subjective emotional experience, symbolic representations, and the psychodynamic themes inferred by the clinicians. This dual-level approach allows for a comprehensive understanding of the client’s internal world as expressed through artistic production.

The procedure of analysis is as follows. After each group session, the art therapist documented the client’s verbal reflections on the artwork, along with the therapist’s clinical observations. During the analytic phase, the art therapist and the clinical psychologist examined both the manifest and latent content. The manifest content included the client’s use of materials and the formal qualities of the artwork, whereas the latent content encompassed the client’s subjective emotional experience and the therapist’s interpretation of the symbolic and psychodynamic elements present in the imagery.

The case studies are structured in accordance with the procedure detailed above and are grouped based on the primary therapeutic effects observed during art therapy interventions.

### Criteria for interpretation

Interpretations of the artworks were guided by the following criteria designed to ensure clinical and methodological rigor. First, visual features of the artworks were not interpreted in isolation but were consistently examined in relation to clients’ verbal reflections, observed emotional responses during the creative process, and the broader clinical context. Second, emphasis was placed on process‑based and longitudinal consistency. Changes in material use, spatial organization, affective tone, and symbolic themes were interpreted primarily as indicators of process rather than as fixed symbolic meanings. Third, interpretations were constrained by theoretical coherence. Analytic conclusions were situated within established conceptual frameworks - particularly trauma theory, mentalization‑based approaches, and neurobiological models of affect regulation and reward. Fourth, interpretive decisions were subject to collaborative clinical reflection. Clinician interpretations were framed as hypotheses rather than definitive explanations and were always contextualized within the client’s lived experience. Taken together, these criteria served to limit over‑interpretation and to ensure that analytic claims remained grounded, transparent, and clinically meaningful.

### Procedure

The case studies are based on two closed art therapy groups, consisting of 8 and 12 sessions respectively, conducted in 2024 and 2025 at a drug outpatient treatment centre in Budapest. Each group included eight adult participants with SUD. Prior to joining the closed groups, participants underwent an initial interview and signed a consent form permitting photographs of their art therapy creations to be used for scientific purposes, with anonymity strictly maintained. No identifying information is disclosed in the case studies. The groups were facilitated by an art therapist under the supervision of a clinical psychologist. The themes of each session focused on recovery and substance use, including topics such as anxiety, difficult emotions, cravings, the inner child, personal needs, the inner critic, and connection. While the themes followed an overall arc, they were also shaped at times by the group’s dynamics.

### Sample

Participants typically engaged in individual psychotherapy and used nonverbal therapy as a complementary approach. Some had completed rehabilitation programs and attended the sessions as part of aftercare, while others had never participated in therapy before making this their first therapeutic experience. The groups were heterogeneous in terms of social status and recovery stage. Some participants (Table 1) were still actively using substances, while others had maintained abstinence for several years. Most had comorbid disorders, including personality disorders (e.g., borderline, mixed types), ADHD, generalised anxiety disorder, bipolar depression, phobias, and panic disorder. Participants ranged in age from 30 to 58, with an average age of 46. All group members voluntarily agreed to participate in art therapy, demonstrating openness and motivation, with no observed resistance to the method.

**Table 1:** Individuals’ characteristics participating in art therapy sessions

Gender	*N*	%
Women	10	63
Men	6	38
**Substances**		
Alcohol	15	94
Pharmaceuticals	5	31
Illicit drugs	5	31
Opiate maintenance treatment	3	19
**Comorbid disorders** (N)	10	63

## Case studies

### Reducing anxiety and shame

During initial interviews, several clients expressed anxiety about participating in group settings and reluctance to speak in front of others. However, during the sessions, it was observed that their artistic creations enabled them to open more easily and speak with greater confidence. Allowing participants to scribble during the opening circle while introducing themselves helped reduce tension and shift focus away from their anxiety. In the closing circle, many reported feeling calmer and more relaxed by the end of the session.

As in any therapeutic process, the rules and structure of the art therapy group are designed to foster a sense of safety. For example, there is a limit on the number of sessions that can be missed; the group meets consistently every two weeks at the same time; each session lasts two hours; and because it is a closed group, the participant composition remains unchanged, which further enhances stability. It is also reassuring that the art therapist consistently addresses breaches of group norms, such as judging others or speaking in generalisations rather than sharing personal feelings.

Anxiety within the group is often reflected in clients’ behaviours. Some seek objects that provide a sense of security - for instance, asking during the opening circle if they may use emotion cards from their bags to help express their feelings (ultimately, they did not use them, but knowing they were available was sufficient). Others check their phones during the creative process to visualise something that emerged in their mind. These behaviours likely stem from anxiety, fear of losing control over the creation, fear of confronting personal emotions, or fear that the artwork might reveal unexpected and difficult content.

The use of structured versus unstructured instructions, depending on the therapeutic goal, can also elicit anxious responses. For some, open-ended tasks may provoke discomfort due to a lack of clear boundaries, while others may feel constrained or overwhelmed by highly specific directives. These emotional reactions and underlying experiences can themselves become valuable material for therapeutic exploration.

Excessive clarifying questions about the instructions - such as “Am I doing it right?” or “Is this how it should be done?” - often reflect anxiety, low self-confidence, and a desire to conform. When given the freedom to choose tools, clients typically gravitate toward familiar materials that provide a sense of security. The balance between freedom and restriction is also evident in the selection of artistic tools.

**Fig. 1:**
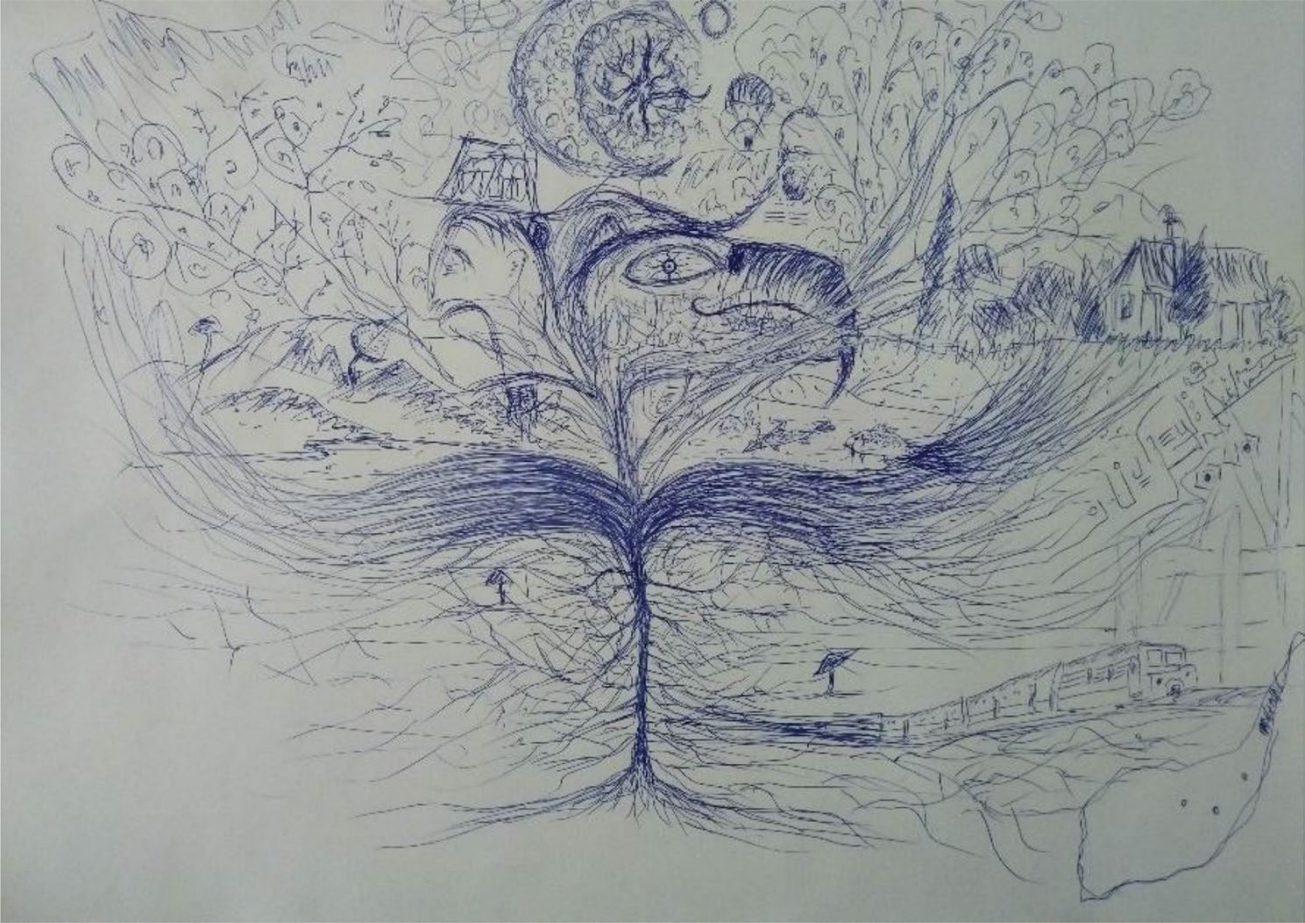
Artwork by a 45-year-old man with SUD/AUD on the theme of “Inner Self-Portrait”

**Fig. 2:**
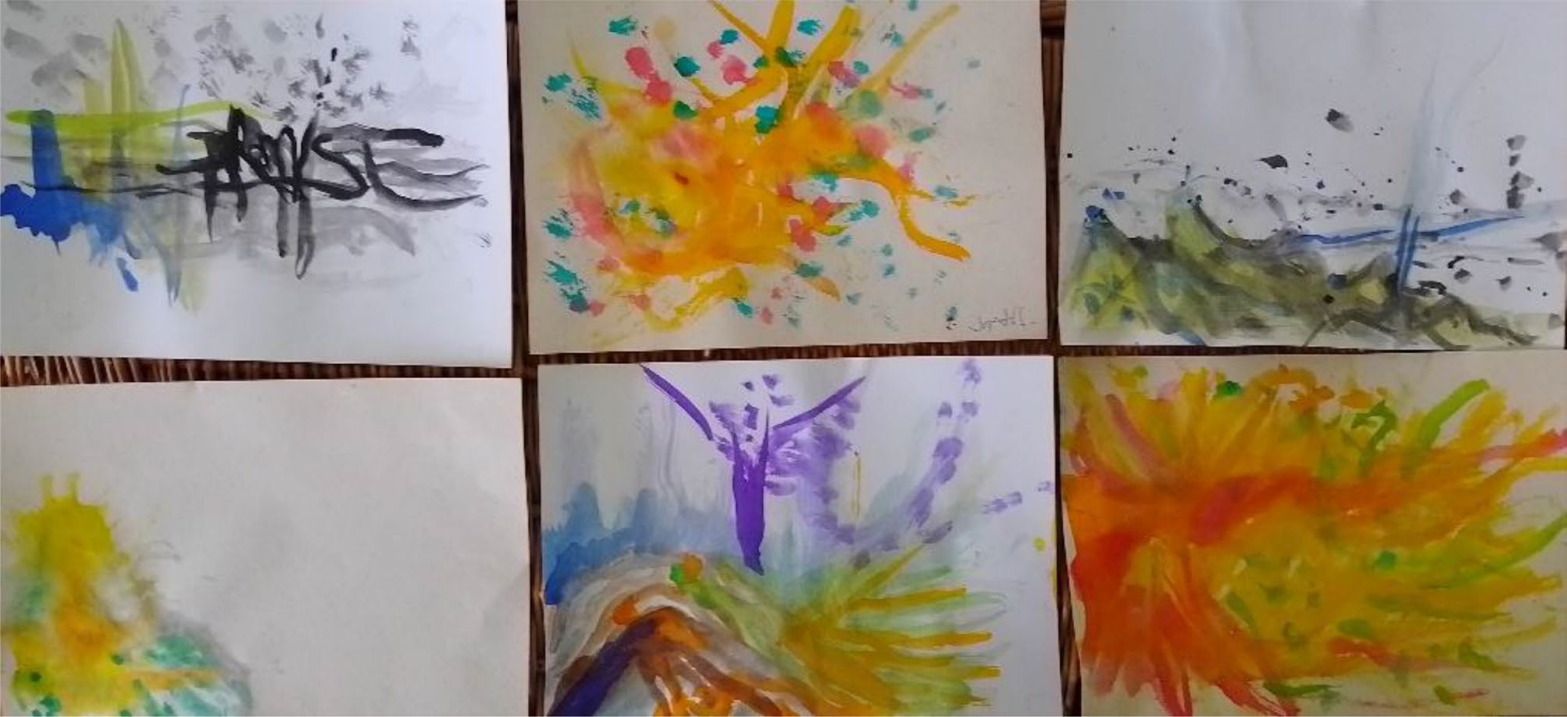
Artwork of a 45-year-old man with SUD/AUD, set to music

For example, a 47-year-old man undergoing opiate maintenance treatment consistently chose pencils and occasionally pastels when given free choice (Figs. 1 and 2). His artworks were meticulously detailed and somewhat compulsively structured, reflecting a need for control and precision. The presented drawing (Fig. 2), executed in blue ink, features a symmetrical composition centered around a tree-like form with extensive, root-like lines spreading downward and branching upward into intricate, organic patterns. The upper section contains swirling, spiral motifs and layered imagery, including abstract shapes and architectural elements such as a house and a tower. The lower section includes linear forms resembling a railway track and small human figures, integrated into a dense network of lines. The overall impression is one of complexity and order, achieved through repetitive, fine strokes and careful spatial organization. The absence of color and reliance on a single medium reinforces the sense of restraint and control. This pattern offers insight into his emotional regulation strategies and underlying psychological dynamics. However, when instructed to use watercolours and paint to music, he was able to relinquish his compulsive tendencies and produce looser, more expressive works (Fig. 1). The directive to paint to music facilitated a shift from cognitive control to embodied emotional expression. This process supports affect regulation by allowing implicit states to surface in a sensory, nonverbal form. The inclusion of text within one painting indicates an integrative step - linking visual and verbal modalities, which enhances reflective functioning [38].

In contrast, the emotion-releasing effect of watercolours did not manifest for a 37-year-old man with alcohol use disorder. Although he painted with watercolours to music, he used the brush as if it were a pencil - his preferred tool - indicating an inability to let go of familiar methods and an unconscious fear of confronting his emotions. This resistance indicates limited capacity for mentalization in the moment - the client remained anchored in familiar, structured methods, avoiding the vulnerability associated with spontaneous creativity.

### Viewing painful memories from a safe distance: Projective surface and emotional regulation

Beyond its anxiety-reducing effects, art therapy offers a projective surface for processing difficult emotions and experiences. By externalising internal critical voices or painful feelings through artistic creation, clients can observe these experiences from a safe psychological distance. This distancing allows the emotions to become more conscious and objectified - no longer experienced as intrinsic parts of the self. Such externalisation facilitates emotional processing and release, while also creating space for unconscious material to emerge and be explored.

**Fig. 3:**
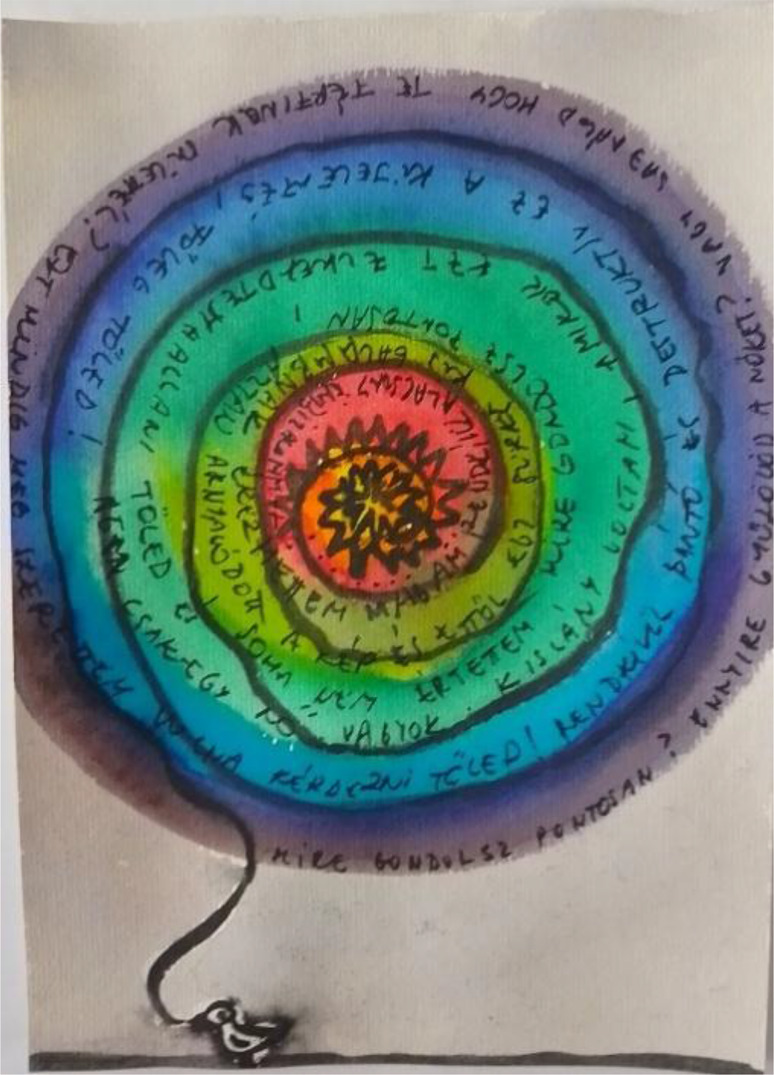
Artwork of a 47-year-old woman with AUD on the theme of “inner critical voice”

The inner critical voice is a significant driver of addiction, making it a crucial focus in the recovery process. One illustrative case involves a 47-year-old woman with alcohol use disorder, who worked with the internalised message “You’re just a woman” - a phrase repeatedly spoken by her father and one that continues to surface in various life situations (Fig. 3). During therapy, she represented this inner critical voice as a bird, which engaged in a symbolic dialogue with her. Through writing, she remained connected to the difficult emotion, gradually reshaping and transforming it. This process often culminated in moments of insight and clarity - what she described as ‘aha’ experiences. By externalizing the critical voice into a tangible image, the client initiated a process of mentalization - transforming implicit affect into a representational form that could be reflected upon and discussed [38]. This shift from “I am worthless” to “This bird represents my father’s voice” illustrates the development of reflective functioning and emotional distancing.

### Supporting the processing of traumatic experiences and exploring unconscious, preverbal mental content

Art therapy facilitates the emergence of traumatic experiences more readily than verbal expression alone. A recurring theme in art therapy sessions is the inner child, highlighting the prevalence of traumatic childhood experiences among individuals with SUD. One illustrative case involves a 30-year-old woman with SUD/AUD who selected a chubby, red-haired, freckled girl from the OH cards, explaining, “You can find something on her appearance that you can tease her about.”

OH cards, created by artist Ely Raman in collaboration with psychotherapist Moritz Egetmeyer, are described as “a door-opener for self-reflection and imagination” and are widely used across therapeutic contexts to support self-exploration and emotional insight [39].

**Fig. 4:**
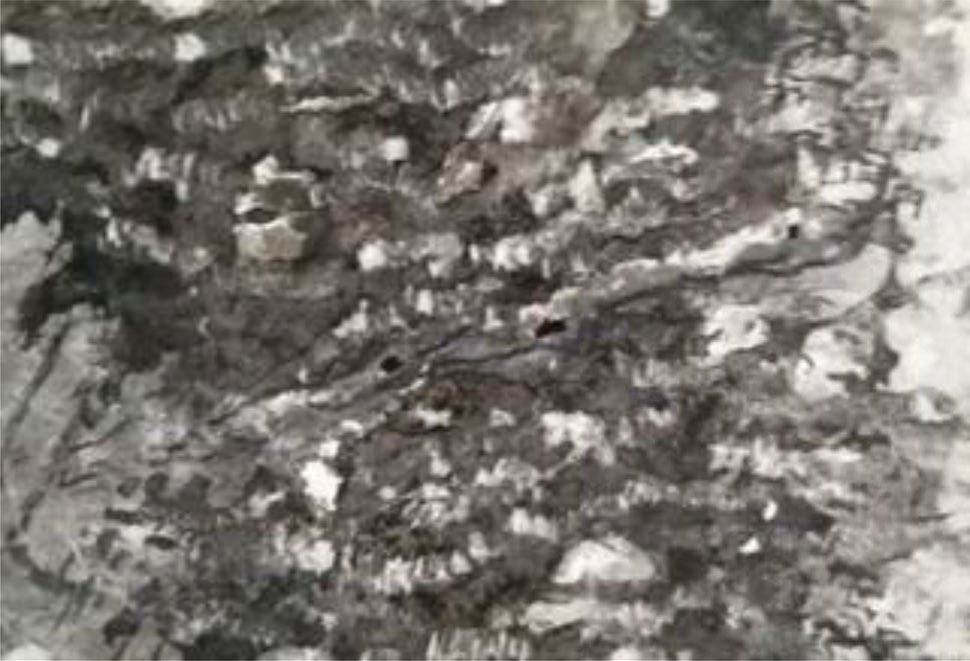
Artwork by a 30-year-old woman with SUD/AUD on the theme of “Inner Child”

The client disclosed a history of ongoing abuse during childhood, including by her brother, while her parents failed to intervene or offer protection. In response to a prompt about what a child might need, she did not depict an idealised or desired family image (Fig. 4). Instead, she created a raw, abstract composition dominated by dark, earthy tones - primarily black, gray, and muted brown. The surface is heavily textured, with layered strokes and irregular patches that evoke turbulence and fragmentation. There is no central figure or organizing structure; instead, the image conveys chaos and density, punctuated by small, indistinct voids that resemble wounds or ruptures. The absence of bright colors or any representational elements suggests an emotional state marked by despair and disconnection rather than hope or idealization. The chaotic, non-representational nature of the artwork reflects the client’s implicit memory of abuse - what van der Kolk et al. [3] describes as preverbal trauma, which often emerges through sensory and somatic channels rather than narrative form. The dense layering and absence of spatial openness may symbolize entrapment and the overwhelming affect associated with chronic victimization. The lack of an idealized family image underscores the dominance of lived experience over unmet needs, consistent with Herman’s concept [25] of trauma-driven identity formation.

During the reflection circle, when asked what might have helped in her abusive situation, she responded, “Understanding and protection from my parents, but they provided financial rather than emotional support.” Her rebellious nature was evident both in group dynamics and in her artwork. This manifested in difficulties with maintaining boundaries, impulse control issues (e.g., a blank, bored expression when receiving instructions, acting out), rejection of conformity (“the familiar is boring”), and a sense of outsider status (e.g., always last to share, silent during association exercises).

**Fig. 5:**
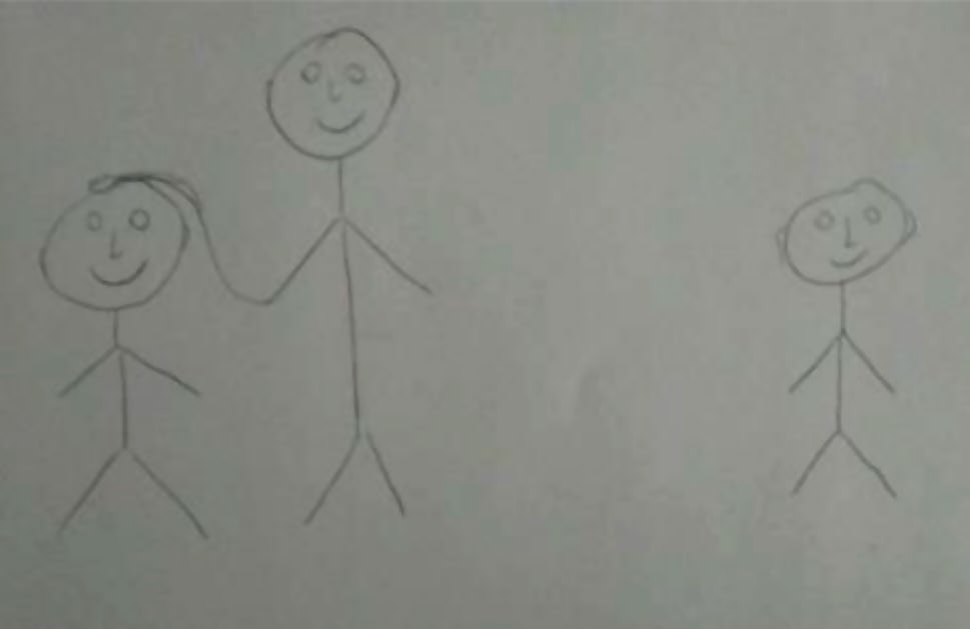
Artwork by a 45-year-old man with AUD on the theme of “Inner Child”

In the case of a 45-year-old man with AUD, a comparison between his artwork on the theme of the inner child and his other creations reveals clear signs of regression in both tool usage and artistic execution (Fig. 5). While his previous art therapy pieces were vibrant, expansive, and filled with colour - often occupying the entire space - his depiction of the inner child stood in stark contrast. In this work, he avoided colour entirely, opting instead for stick figures surrounded by space. This visual language reflected emotional distance, vulnerability, and a possible disconnection from early experiences. The composition consists of three stick figures: two positioned close together on the left side, and one isolated figure on the right. The figures are rendered with simple circular heads and straight lines for limbs, without any color or background detail. The left pair includes a taller figure with an arm extended toward the smaller figure, suggesting a gesture of care or protection. The solitary figure on the right stands apart, occupying empty space, which dominates the majority of the page.

The use of pencils, which often evoke a sense of security and are associated with childhood, alongside simple, childlike lines, marked a significant departure from his earlier, more expressive and colourful works. A photograph of the client as a primary school boy, approximately 10 years old, was used during the session. He engaged deeply in the first round of the exercise, as numerous memories surfaced - yet he was unable to share them verbally. He acknowledged the need to confront these memories, but doing so caused him considerable emotional distress.

During the reflection circle, the client explained that his artwork represented what he had longed for as a child but never received. He depicted two children: one standing alone, symbolising freedom, and another standing beside a supportive adult, though he did not specify who this figure represented. In this case, regression was evident both in the artwork and in the reflection process, characterised by a rapid shift from emotional engagement to suppression. The regression to childlike drawing forms and the absence of color indicate a reactivation of early attachment-related trauma. The isolated figure may symbolize the client’s experience of emotional abandonment, while the paired figures represent an unmet longing for protection and relational security [3]. Nonetheless, signs of self-reflection and mature avoidance - such as the use of humour - were also present, indicating a complex interplay between emotional vulnerability and coping strategies.

**Fig. 6:**
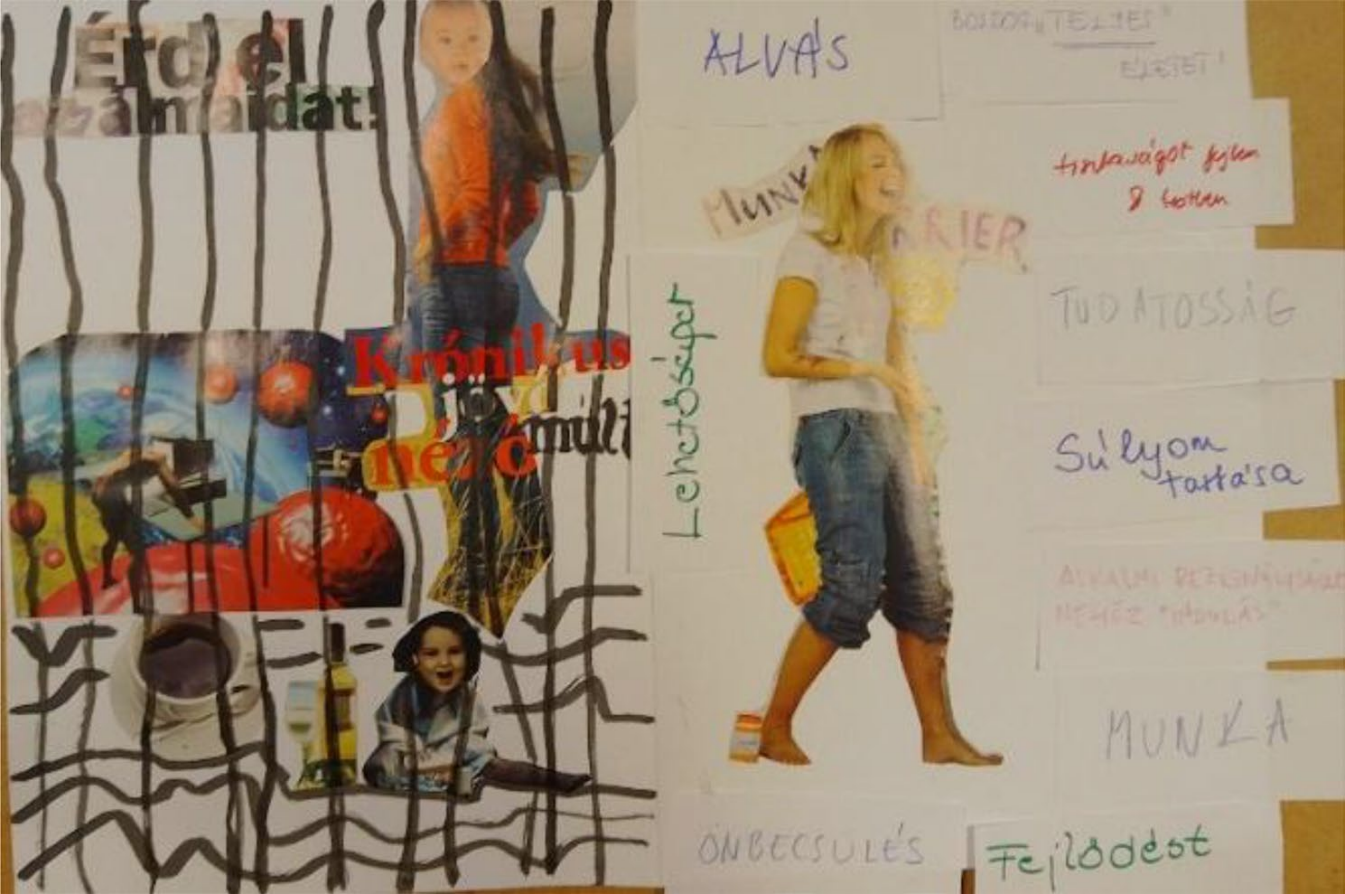
Artwork by a 47-year-old woman with AUD on the theme of “substance use identity and substance-free identity”

Art therapy is a highly effective method for identifying personal resources. Clients often express a strong preference for the montage technique, which - despite initial reservations as a group leader, which originate from concerns that it might limit cognitive engagement - has proven to be both enjoyable and empowering for participants. In practice, clients frequently regard their montage-based creations as the most rewarding, and sessions utilising this technique consistently have a positive, energising impact. When exploring the theme of substance-using versus substance-free identity, the montage technique has been particularly helpful in facilitating self-expression and in illuminating the contrast between these two aspects of identity (Fig. 6). The selected montage is divided into two distinct sections, visually and symbolically contrasting two identity states: substance-using versus substance-free. Left side (substance-using identity): Dominated by dark, heavy vertical lines resembling prison bars, which create a sense of confinement and restriction. Behind these bars are images associated with addiction - alcohol bottles, pills, and fragmented text such as “Krónikus” (chronic) and “Éld az álmaidat” (live your dreams - ironically juxtaposed with the reality of dependency). The chaotic arrangement and overlapping elements evoke disorganization and loss of control. Right side (substance-free identity): Features a more open, structured composition with a central full-body figure walking forward, symbolizing movement and progress. Surrounding this figure are handwritten words and phrases such as “Lehetőség” (opportunity), “Önbecsülés” (self-esteem), “Fejlődés” (growth), and “Munka” (work), reflecting aspirations and values aligned with recovery. The lighter background and absence of restrictive lines convey freedom and clarity. The creative process may itself activate reward pathways, offering a non-substance-based source of gratification. The energizing impact of montage, as reported by the client, aligns with findings that art-making can substitute maladaptive reward systems with adaptive, embodied experiences of agency and control [5, 30].

### Less easy to manipulate

As a nonverbal therapeutic method, art therapy elicits less psychological resistance and is significantly more difficult to manipulate than verbal communication. In art therapy groups, a striking contrast has been observed between clients’ verbal expressions and the content of their artwork.

One example involves a 50-year-old man with AUD who initially expressed a desire to work with watercolours. However, when given free choice of materials, he consistently selected pencils. This behaviour likely reflected an unconscious need to engage with his emotions in a controlled manner. Although he claimed that watercolours were “too messy,” it is more plausible that he was not yet emotionally prepared to confront what his emptiness concealed. The pencil, familiar and stable, offered a sense of safety - it did not slip and provided a solid foundation.

Throughout the sessions, he remained largely silent about his emotions, often responding with a tense “Everything’s fine” and maintaining a withdrawn, taciturn demeanour. His artworks featured large empty spaces and schematic representations, suggesting symptoms of depression and anxiety that contradicted his verbal assertions.

**Fig. 7:**
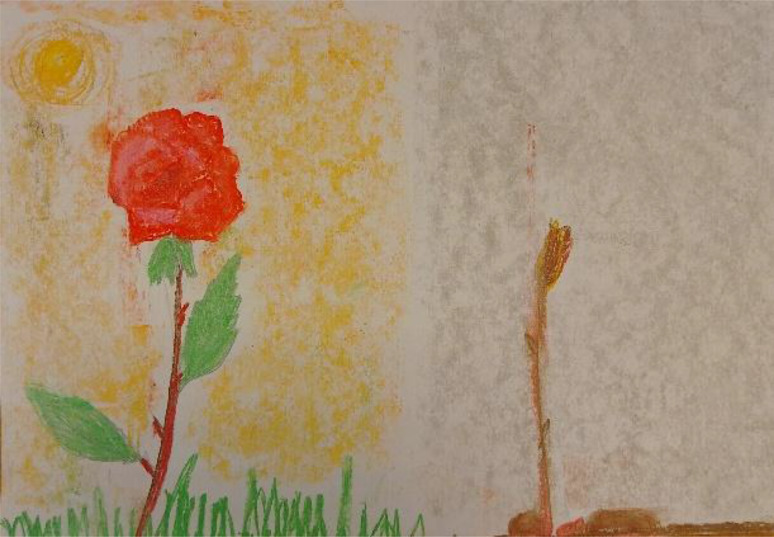
Artwork of a 54-year-old woman with SUD on the theme of “drug-using identity and drug-free identity”

Another case involved a 54-year-old woman with SUD. During the initial phase of therapy, she repeatedly stated that she was happy and that everything was fine, identifying methadone as her only concern. However, when working on the theme of identity with and without drug use, she created an artwork depicting two flowers (Fig. 7): the flower on the left represented her past, when she was drug-free, and the flower on the right symbolised her present, during methadone treatment. The contrast between her verbal claims of well-being and the lifeless flower representing her current state poignantly illustrated the disconnect between spoken words and emotional reality. The idealization of the drug-using identity as vibrant and flourishing may reflect the client’s reliance on substances as a coping mechanism for unresolved trauma. The lifeless representation of sobriety suggests an unconscious fear of emptiness and emotional pain without chemical support. By externalizing these contrasting identities, the client initiated a process of mentalization - transforming implicit affective states into symbolic form. The stark visual contrast may enable her to verbalize ambivalence about recovery, bridging the gap between unconscious fears and conscious reflection.

### Producing something of value: a self-confidence booster

**Fig. 8:**
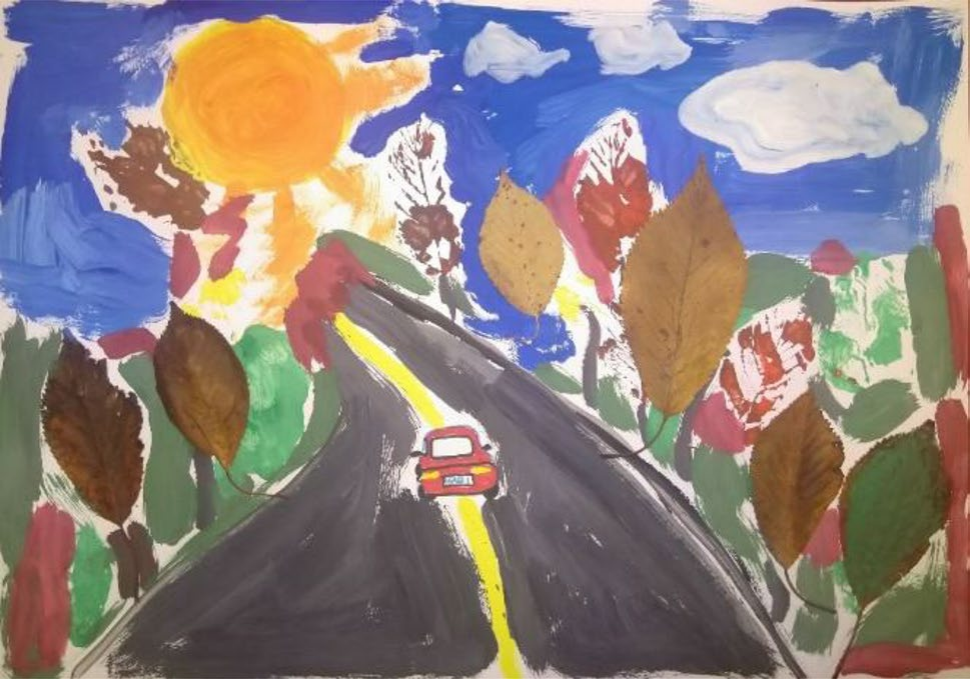
Artwork of a 50-year-old woman with SUD/AUD on the theme of “autumn tree visualisation”

Art therapy offers the joy of creating something meaningful, allowing individuals to experience their own creativity independent of substance use. This process fosters a sense of accomplishment and strengthens self-confidence. One notable example involves a 50-year-old woman with SUD/AUD. The act of creation during therapy significantly boosted her self-esteem and reaffirmed her belief in the value of pursuing artistic expression. As a testament to its personal significance, she chose to hang her artwork on the wall at home (Fig. 8). The painting depicts a vivid autumn landscape with a central road leading toward the horizon. A red car travels along the road, symbolically positioned on a bright yellow dividing line, suggesting movement and forward progression. The background features a large, radiant sun in the upper left corner, casting warm tones across the sky, which transitions into deep blue with scattered white clouds. Surrounding the road are trees adorned with real pressed leaves, integrated into the painted composition - a mixed-media approach that adds texture and depth. The overall color palette is vibrant, dominated by warm oranges, reds, and greens, evoking vitality and optimism. The perspective of the road converging toward the distance creates a sense of journey and future orientation. The creative process - particularly the integration of natural elements (pressed leaves) - likely activated dopaminergic reward pathways, offering a pleasurable, non-substance-based experience. This engagement provided a sense of mastery and accomplishment, essential for sustaining motivation in recovery.

### Objectification of emotional experiences

Unlike verbal therapy, art therapy produces tangible creations that exist in space and time - objectified emotional expressions that can be revisited and reintegrated into the therapeutic process. These artworks serve as lasting representations of internal states, allowing clients to reflect on their emotional journeys over time.

**Fig. 9:**
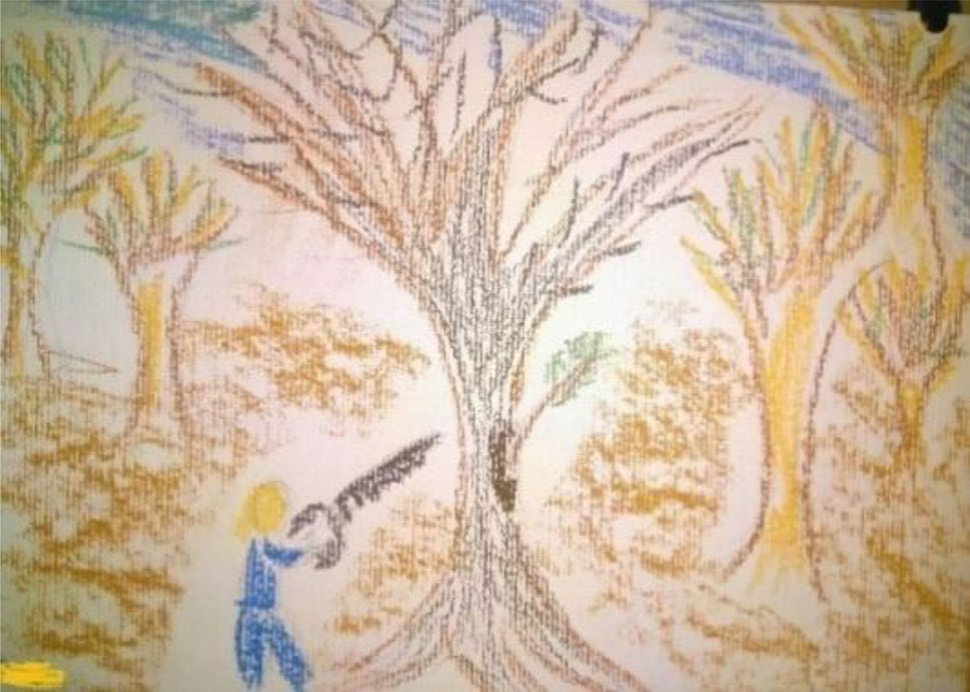
Artwork of a 57-year-old woman with AUD on the theme of “autumn tree visualisation”

One compelling example involves a 57-year-old woman with SUD/AUD. When her artworks (Fig. 9) were displayed in chronological order, she was astonished to discover that the first tree she had painted was her own - she had no recollection of creating it. At the time she began therapy, she was experiencing severe depression and suicidal ideation. In retrospect, she recognised that the lifeless tree depicted in the artwork mirrored her emotional state during that period. Additionally, at the base of the tree, a small figure in blue appears to be cutting into the trunk with a saw, suggesting an act of destruction or transformation. The barren tree and act of cutting may symbolize the client’s internal experience of fragmentation and self-directed aggression, common in trauma survivors and those struggling with severe depression. The absence of foliage reflects emotional depletion, while the tiny green shoot represents a nascent capacity for recovery - a visual metaphor for resilience emerging from despair.

Two significant therapeutic moments were associated with this piece. Initially, her painful emotions surfaced nonverbally - she was unable to articulate them. It was only three months later, upon revisiting the artwork, that she found the words to describe what she had felt. This case underscores the importance of preserving and periodically revisiting creative works in therapy. New insights may emerge over time, and looking back can facilitate emotional release and help clients let go of experiences that no longer define them. This case might demonstrate the therapeutic value of reflection and the gradual development of mentalization.

### Supportive and safe group environment

**Fig. 10:**
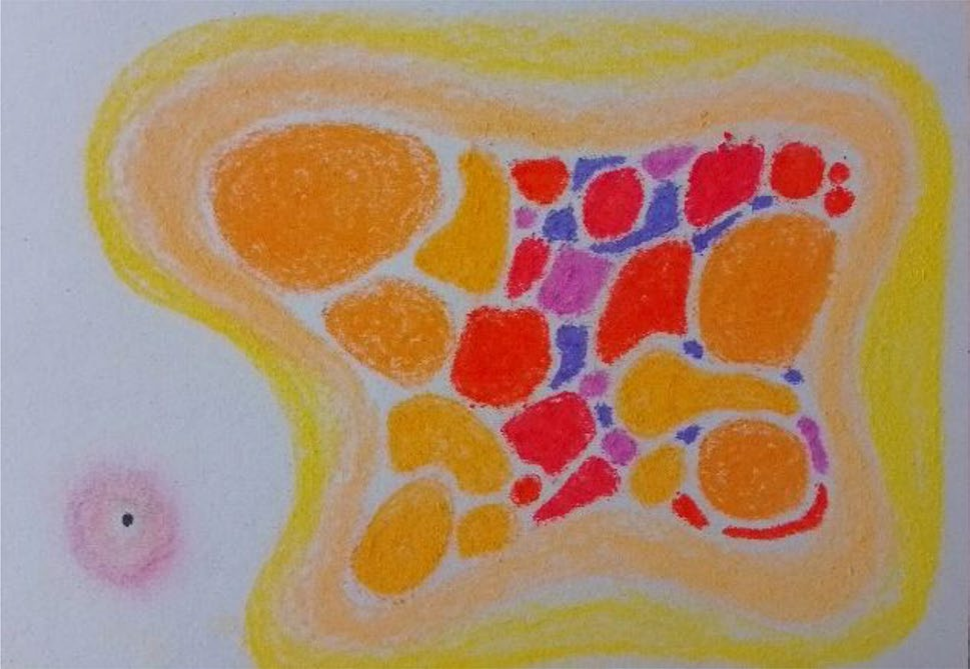
Artwork of a 32-year-old woman with AUD on the theme of “me and others”

**Fig. 11:**
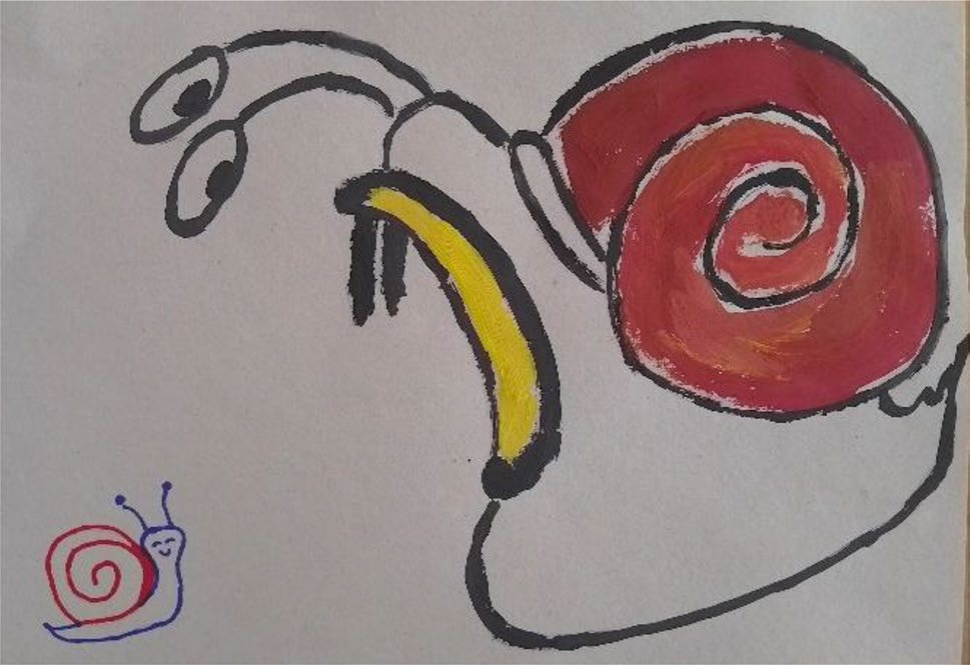
Artwork of a 50-year-old woman with SUD on the theme of “me and others”

Art therapy in a group setting enables clients to experience connection through their creative work within a supportive and safe environment - often one they have never encountered before. Many participants reported feelings of loneliness and isolation, and lacked close, intimate relationships. This emotional isolation was clearly reflected in the artworks created in response to the theme “Me and Others.” (Figs. 10 and 11). For example, in the case of 10. Figure, the composition features two distinct elements: on the left side, a small, dark dot surrounded by a faint pink halo, positioned in isolation on a white background. On the right side: A large, irregular cluster composed of overlapping organic shapes in warm tones - orange, yellow, and red - with smaller purple and pink forms interspersed. The cluster is encircled by layered outlines in peach and yellow, creating a sense of cohesion and density. The stark spatial separation between the solitary dot and the vibrant cluster conveys a powerful visual metaphor for relational distance and emotional isolation. This contrast enabled reflective dialogue about belonging and exclusion, supporting affect regulation and interpersonal awareness.

Similarly, another piece (Fig. 11) depicted a small dot and a snail on the left side, representing the client, while a large abstract circle and a larger snail on the right side symbolised others. The exaggerated size and strong color contrast convey dominance and presence. The stark difference in size and complexity between the two snails creates a visual metaphor for relational imbalance and perceived insignificance.

Such visual representations of loneliness were consistent across multiple artworks. Importantly, clients were able to recognise that they were not alone in experiencing these feelings. This realisation had a significant anxiety-reducing effect and contributed to enhanced group cohesion, underscoring the therapeutic power of social support within the group context.

### Group dynamics: Collaborative creation - fostering connection and group cohesion

For a 50-year-old woman with SUD struggling with relationship difficulties, the experience of creating a joint artwork was one of the most liberating moments in therapy. The collaborative painting exercise began with an imaginative prompt: clients were invited to depict whatever emerged in their minds on a large, shared sheet of paper. Following this individual phase, they were asked to develop a unified group image together.

Initially, the client created her own image, then stood up and refrained from continuing. In the reflection circle, she later explained that she felt unable to connect with the individuals sitting beside and across from her. Eventually, she returned to the artwork and, together with the client seated to her right, co-created a mountain - an act she described as deeply liberating.

**Fig. 12:**
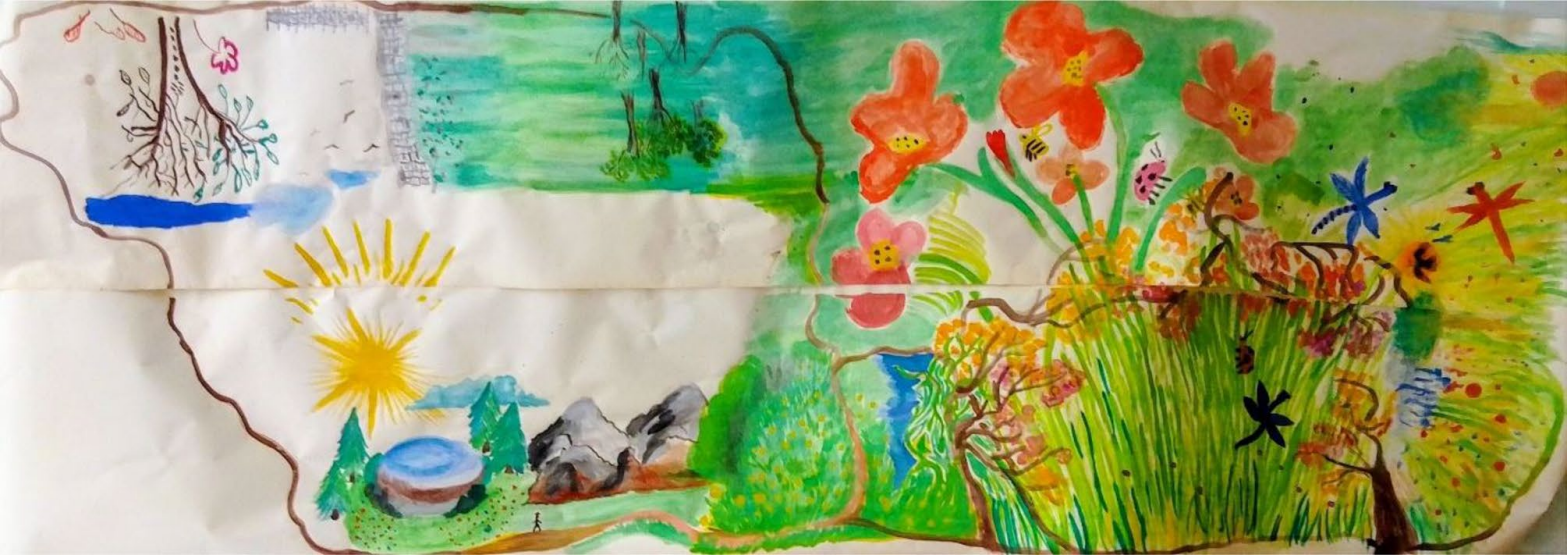
Joint painting

The final joint painting visually reflected the evolving group dynamics (Fig. 12). The large, horizontal composition is a vibrant, multi-sectioned painting. Left section features a tree with exposed roots, a small blue pond, and a stone-like structure, suggesting grounding and stability. Central section includes a radiant yellow sun above green hills and a circular blue element resembling water or a vessel, symbolizing life and renewal. Nearby, gray rocks and a mountain motif indicate strength and resilience. Right section is dominated by lush greenery and vivid orange-red flowers, interspersed with bees and dragonflies, evoking vitality and interconnectedness. The layering of colors and overlapping elements reflects active engagement and integration among participants. Despite the overall harmony, subtle gaps remain between certain sections, visually representing relational distance for clients who struggled to connect fully. The process of negotiating shared space and integrating individual contributions fostered mentalization - clients reflected on their own and others’ intentions, enhancing interpersonal awareness and emotional regulation.

Connections were clearly established between clients seated next to and opposite each other. Some participants engaged more boldly, integrating their work with others’, while others remained more reserved. Notably, a visible gap persisted between two or three clients who were unable to connect through the artwork.

During individual consultations, these relational gaps were explored further, focusing on the emotions that arose and the underlying reasons why connection could not be established. The joint painting revealed unspoken yet palpable relational tensions within the group, as well as the communication challenges some clients faced in verbal interactions.

Despite these difficulties, the act of creating together - through the nonverbal medium of art - enabled clients to connect, collaborate, and produce a shared work. This process helped reduce feelings of isolation and fostered a sense of group cohesion, demonstrating the therapeutic power of collective artistic expression. In addition to that, joint creative engagement reinforced positive social interaction and reduced anxiety. The sensory richness of the artwork - bright colors, organic forms - likely contributed to dopaminergic activation, promoting feelings of pleasure and safety.

## Discussion

The case studies presented in this research reaffirm findings previously established in the literature: art therapy is an effective modality for individuals with SUD and plays a vital role in their care. The integration of art into addiction treatment is supported by scientific evidence. Neuroscience continues to expand our understanding of how the brain and body respond to stress and trauma, and how images and creative processes influence emotional, cognitive, and physiological well-being. Research confirms that the visual, sensory, and expressive language of art can be effectively incorporated into therapeutic interventions [7, 40]. The nonverbal nature of artistic expression further facilitates access to pre-mentalization states, enabling clients to externalise and process affective experiences that may otherwise remain inaccessible [38].

Numerous cases confirm that creativity itself has a healing and cathartic effect, particularly in reducing stress and anxiety [41]. Art therapy supports the development of self-awareness and assists in processing traumatic experiences. It allows painful memories to be viewed from a safe distance and through a new perspective, facilitating the exploration and externalisation of unconscious, preverbal psychological content. The act of creation promotes emotional regulation and self-control, while the production of meaningful artwork fosters self-confidence. Nonverbal techniques are less susceptible to conscious manipulation and reduce psychological resistance. Moreover, the tangible, permanent, and spatial nature of the artwork enables the objectification of emotional experiences. Within a supportive and safe group environment, clients may experience a sense of connection that they have not previously encountered.

The group setting demonstrated the power of community, revealing participants’ capacity and willingness to connect through creative expression - even among those who were typically reserved or anxious in social contexts. The creative process enabled clients to feel acknowledged and understood.

The regression elicited by the inner child theme supported existing literature indicating that deep traumas and shame surface more readily through nonverbal means than through verbal communication [42]. Difficult emotions such as anxiety and suicidal ideation, initially expressed through artwork, were later integrated into the therapeutic process and became verbalizable. The artwork provided both emotional safety and distance from the traumatic experience, while still allowing the trauma to be expressed and processed. Furthermore, the case studies confirmed the therapeutic value of revisiting artwork over time, as clients often gained new insights upon reflection.

The use of artistic materials also played a role in dismantling defence mechanisms and reducing excessive control. While tools such as pastels and pencils often aligned with intellectualising tendencies - encouraging overthinking and meticulous detailing - the exclusive use of watercolours challenged these patterns. With less capacity for precision, clients produced more spontaneous and emotionally expressive works.

Art therapy proved less manipulable than verbal therapy. Emotional states such as anxiety, depression, and emptiness were clearly reflected in the formal characteristics of the artwork and choice of tools, often contrasting with verbal expressions like “Everything is fine.” In several cases, clients were able to identify personal resources and reconnect with their creativity through the artistic process.

The artwork served as a projective surface, allowing negative emotions and internal critical voices to be transformed into visual form. Difficult feelings continued to be processed and reshaped through complementary activities such as letter writing, often culminating in moments of insight or “aha” experiences.

The creative process helped clients experience control, self-regulation, and a sense of competence, independence, and agency - factors that contributed to improved self-assessment [43]. It also had an empowering and confidence-building effect, particularly through goal identification, resource recognition, and feedback received during reflection rounds. Joint creation fostered a sense of connection and cohesion, and the group-based format of art therapy was especially effective in promoting interpersonal engagement among clients who initially exhibited social withdrawal and anxiety. The shared creative process bridged isolated inner experiences and communal expression, reducing alienation and enhancing group cohesion.

Reflection rounds were consistently valuable and well-received. Clients expressed gratitude for peer feedback, which visibly reinforced their sense of connection. For some, the reflection circle provided insight into their stage of recovery. Typical symptoms associated with addiction - such as cognitive distortions (e.g., “must” and “have to” thinking), impulse control disorders, acting out, emotional emptiness or overload, anxiety, boundary issues, childhood trauma, abuse, and emotional neglect - were evident both in the artwork and during group discussions.

## Limitations

It is important to acknowledge the limitations of art therapy and to avoid overgeneralizing its therapeutic effects. While the literature – including the empirical findings of randomized controlled trials - documents a range of potential benefits in reducing various psychiatric symptoms [44, 45], these effects are neither universal nor uniformly expressed across individuals, diagnostic groups, or treatment phases. Art therapy does not constitute a stand‑alone intervention, nor should it be assumed to produce rapid or overt symptom change in all cases [2]. Therapeutic change in art therapy often unfolds slowly, through shifts in emotional awareness, self‑regulation, or meaning‑making rather than through immediate symptom reduction [46, 47]. Such processes are comparable to the incremental changes observed in other psychotherapeutic modalities and should be interpreted accordingly. Consequently, claims regarding the effectiveness of art therapy should be situated within a nuanced, integrative framework that recognizes both its strengths and its inherent constraints.

Furthermore, our findings were based on qualitative clinical case material derived from a limited number of art therapy groups, which restricts generalizability. Additionally, the study did not include standardized quantitative outcome measures, such as symptom severity scales, client-rated change indices, or clinician-administered assessments. As a result, the reported therapeutic effects cannot be interpreted as evidence of efficacy in a statistical sense. Instead, the findings should be understood as process-oriented clinical observations that illustrate how therapeutic change may unfold through artistic expression in addiction treatment.

## Clinical implications and future directions

These findings underscore the importance of incorporating structured yet flexible art therapy interventions into multidisciplinary addiction treatment programs. Given the high prevalence of comorbid personality disorders and trauma histories among individuals with SUD, art therapy can function as an effective complement to verbal psychotherapy and pharmacotherapy by engaging therapeutic processes through a different modality.

Future research should explore longitudinal outcomes, including relapse prevention, emotional resilience, and shifts in self-concept, to further validate the long-term efficacy of art therapy in addiction recovery.

Art therapy is also an excellent tool for status assessment, offering insights into clients’ personalities and emotional landscapes. The case study findings, consistent with existing literature, confirm that art therapy is highly suitable for use with patients with SUD. The healing power and supportive nature of creativity provide a safe space for exploration. Art therapy reveals information about personality, change, and behavioural patterns; it indicates where clients are in their recovery and self-awareness; it highlights obstacles and challenges; and it offers numerous therapeutic benefits.

## Data Availability

Not applicable.

## Declarations

### Authors’ information

PA holds a PhD and is an art therapist, sociologist, and addiction consultant. They serve as a research fellow in a university addiction department and work as an art therapist in a drug treatment centre. MKF holds a PhD and works as a clinical psychologist at a drug treatment centre, as well as an associate professor in a university addiction department.
